# Digitaline

**Published:** 1851-11

**Authors:** 


*Digitaline.*—Some highly interesting researches on *digitaline,* the active principle of digitalis, have been recently made in France, by MM. Quevenne and Homolle. The following are the chief points of interest which they present:—

"Digitaline is soluble in small quantity in the serum of the blood, without producing in it any apparent change. The proportion dissolved appears to be the same as in water. It is dissolved also in filtered gastric juice. Digitaline exerts no action upon litmus. It is dissolved also in small quantity in water; and in all proportions in weak or concentrated alcohol. *The characteristic and distinctive property of digitaline is, to be colored of an emerald green by concentrated hydrochloric acid, forming a turbid solution.* Digitaline belongs to the class of immediate neutral principles; it combines neither with acids (the tannic excepted) nor with alkalis.

"To be assured of the *identity* or of the *quality* of digitaline, an important and capital point in a therapeutical view, MM. Homolle and Quevenne have paid attention to the different degrees of bitterness of this substance; for they state that its degree of bitterness is incontestably a measure of its degree of energy. To succeed in this test, the following plan is to be adopted: 'One centigramme of digitaline is dissolved in two grammes of alcohol, and the mixture is progressively diluted with water, until the complete disappearance of the bitter taste. If the digitaline is good, this quantity will require the employment of two *litres* of water.' This method of appreciating the intensity of the taste of bodies, employed already by other observers, to judge of the richness of certain medicinal preparations, has been considered by MM. Homolle and Quevenne as proper for enabling them to obtain digitaline always identical with itself. This test is the more indispensable, because, in conforming rigorously to the mode of preparation just described, no one can be certain of having always the same product. Digitaline, except in its action on the animal economy, does not possess, unfortunately, any essential property by which we can positively recognise its purity. It does not crystallise, and does not form any crystallisable combinations; we can therefore never affirm that it has been obtained sufficiently pure, or that it has not been mixed. Let the person who prepares digitaline, therefore, be well acquainted with the fact, that this product does not offer any suitable proofs of its purity, except inasmuch as, subject to the experiment of dilution, it gives the same result as a *type* of acknowledged purity. Let the physician be also warned that digitaline does not constitute a medicine of such a certain identity that he should neglect a great degree of prudence in its use; a prudence which, besides, is demanded by the extreme activity of this new therapeutic agent.

"MM. Homolle and Quevenne having thus succeeded in obtaining digitaline in the state which they call *identical*, it remained for them to determine what was the pharmaceutic form for giving it, under the quadruple relation of *safety*, *convenience*, *good preservation*, and *easy administration*. The solution of this problem was the more important, because the product under consideration possesses a very great degree of activity, so that it ought to be administered only in milligrammes, or, in fact in an *infinitesimal* dose. Among the different pharmaceutical forms considered by MM. Homolle and Quevenne, the pilular form is that to which they have given the preference. 'The variety,' say these authors, 'which appears to us the best, is that of little sugar-plums, in which the active matter is enveloped in a layer of sugar, so that the patient, by reason of their very small size, can swallow them easily, without mastication, and therefore without any other perception than that of the sugar which covers them.' These sugar plums are called granules by the authors, and each contains a milligramme of digitaline; and it appears that this mode of administration preserves the powers of the drug to a period of which MM. Homolle and Quevenne have not ascertained the limit.

"As regards the physiological and therapeutic action of digitaline, one of the authors made a series of experiments on himself, and his exemplary exactness in recording the results is warmly eulogised by the reporters. From the analysis of the first six trials, undertaken in the course of the years 1842, 1843, 1847, 1848, 1850, it results that the *medium* of the diminuition in number of the pulsations of the heart and arteries has been about four pulsations *during*, and four *after*, the administration of the medicine. Experiments made on two dogs proved that, in one, the pulse was lowered from 59 to 51, and that in the other, from 87 to 69, by the administration of minute doses of digitaline. As to the comparative efficacy of the preparations of digitalis hitherto employed, and of digitaline, MM. Homolle and Quevenne leave this point to be finally determined by clinical experience; but they think that their own observations, and the works published up to the present time, lead to the belief that the question will be resolved in favor of digitaline; and they quote the authority of MM. Hervieux, Strohl, and Sandras, who have all employed the digitaline in their practice with most happy results. M. Hervieux states, that one of the circumstances which plead most in favor of digitaline, is the facility of apportioning the dose; for, while this can be done in the case of the preparations of digitalis only by a very rough approximation, it can be accomplished in the case of digitaline with a mathematical rigor. M. Sandras declares, that he has never met with the inconvenience attributed to digitalis, while he has employed digitaline; the latter has appeared to him to be easy to take, and easy for the patient to bear; its effect has been perfectly fixed and regular, according to the doses in which he prescribed it. Some clinical observations made by MM. Homolle and Quevenne are then related, but we cannot say either that the cases seem to be well chosen, or the results accurately noted; and the indiscriminate manner in which the digitaline was prescribed, is not to be commended. In the first case, for instance, there was a 'puerperal affection,' but we are not told what was its nature; in the second, there was an 'affection of the heart,' and a 'profound disturbance of the circulation;' in the third case, there was hypertrophy of the heart, with 'lesion of the orifices;' in the fourth case, there was 'profound perturbation of the cardiac circulation;' all these terms are far too vague for the present advanced state of science. Again, the digitaline was given in the seventh case for cyanosis, from persistence of the foramen ovale; and in the sixth, it was given in large doses for anasarca with albuminous urine. In our humble opinion, neither of these diseases would justify the use of digitalis in any form.

"The following statement by the authors is of extreme importance, and completely accounts for the frequent uncertainty which attends the employment of digitalis in medical practice. 'We have been able,' say MM. Homolle and Quevenne, 'to ascertain, when we have procured our specimens of digitalis, that, in the quantity which existed in 1847 on the Place de Paris, there was about a fourth part well dried, a fourth of middling quality, and half which was badly dried and manifestly bad. It is evident that this last and considerable fraction was not thrown away; and, as digitalis has only a medical use, we are obliged to admit that it has been consumed by the sick.'"—*London Journ. of Medicine.*

